# Genotyping Analysis for the 46 C/T Polymorphism of Coagulation Factor XII and the Involvement of Factor XII Activity in Patients with Recurrent Pregnancy Loss

**DOI:** 10.1371/journal.pone.0114452

**Published:** 2014-12-09

**Authors:** Eriko Asano, Takeshi Ebara, Chisato Yamada-Namikawa, Tamao Kitaori, Nobuhiro Suzumori, Kinue Katano, Yasuhiko Ozaki, Makoto Nakanishi, Mayumi Sugiura-Ogasawara

**Affiliations:** 1 Department of Obstetrics and Gynecology, Nagoya City University, Graduate School of Medical Sciences, Nagoya, Japan; 2 Occupational and Environmental Health, Nagoya City University, Graduate School of Medical Sciences, Nagoya, Japan; 3 Department of Biochemistry II, Nagoya City University, Graduate School of Medical Sciences, Nagoya, Japan; University of Sydney, Australia

## Abstract

**Background:**

Established causes of recurrent pregnancy loss (RPL) include antiphospholipid syndrome, uterine anomalies, parental chromosomal abnormalities, particularly translocations and abnormal embryonic karyotype. A systematic review concluded that coagulation factor XII (FXII) deficiency was associated with RPL. However, it could not be established whether the 46 C/T SNP of FXII or low activity of FXII was a risk factor for RPL, because of the small sample size.

**Methods and Findings:**

We conducted a cross-sectional and cohort study in 279 patients with two or more unexplained consecutive pregnancy losses and 100 fertile women. The association between the lupus anticoagulant (LA) activity and FXII activity was examined. The frequency of the CC, CT and TT genotypes and the FXII activity were also compared between the patients and controls. Subsequent miscarriage rates among the CC, CT, TT genotypes and according to the FXII activity was examined. LA was associated with reduced FXII activity. The CT, but not the TT, genotype was confirmed to be a risk factor for RPL in the cross-sectional study using multivariate logistic regression analysis (OR, 2.8; 95% CI, 1.37–5.85). The plasma FXII activity in the patients was similar to that in the controls. Neither low FXII activity nor the CT genotype predicted the subsequent pregnancy outcome in the cohort study. On the other hand, and intermediate FXII activity level of 85–101% was predictive of subsequent miscarriage.

**Conclusions:**

Low FXII activity was not associated with RPL. The FXII gene was found to be one of the significant susceptibility genes for RPL, similar to the FV Leiden mutation. However, the clinical influence of the CT genotype might be relatively small, because the presence/absence of this genotype did not have any predictive value for the subsequent pregnancy outcome. This was the first study indicating the influence of *FXII 46C/T* on further pregnancy outcomes.

## Introduction

Recurrent miscarriage (RM) is classically defined as three or more consecutive miscarriage [1]. However, many researchers have now revised the definition to two or more pregnancy losses, namely recurrent pregnancy loss (RPL), because of the recent increase in the prevalence of childless couples. The estimated incidence of RM and RPL are 1% and 5%, respectively [1]. Established causes of RPL include antiphospholipid syndrome (APS), uterine anomalies, and chromosomal abnormalities, particularly translocations, in either partner [1]–[4]. However, according to reports, in about a half of the cases seen at research centers, the cause remains unexplained despite conventional examinations conducted to identify the cause [5], [6]. Recently, we found that an abnormal embryonic karyotype was the most frequent cause of 2 or more RPL, accounting for as high as 41% of all the cases [7].

APS, acquired thrombophilia, is the only one treatable cause of RPL, and combined low-dose aspirin and heparin treatment having been shown to improve the live birth rate in patients with APS [8], [9]. Heritable thrombophilia has been reported to be associated with RM [10], [11].

Coagulation factor XII (FXII) is an 80-kDa serine protease that is involved in the initiation of the intrinsic pathway of the coagulation cascade. It is converted to its active form (activated factor XII, XIIa) by limited proteolysis [12], either by autoactivation on the surface of negatively charged compounds or by kallikrein [13]. Although FXII deficiency is associated with a prolonged activated partial thromboplastin time (aPTT), it is not associated with increased bleeding [14]. A C/T polymorphism has been identified in the promoter region of the *FXII* gene at nt46. The 46C/T polymorphism creates a new initiation codon (ATG) for transcription of the mRNA and a frameshift that produces a truncated protein. The T allele destroys the Kozak's consensus sequence (GCCAGCCATGG) for translation initiation signaling and prevents proper recognition of the translation initiation site. The T allele is therefore well-known to be associated with low plasma levels of factor XII [15]. The existence of associations between low FXII activity levels and thrombotic outcomes has been under debate for more than a decade.

We previously reported that the miscarriage rate of patients with low FXII activity (less than 39%) was significantly higher than that of patients with normal FXII activity [16]. We also found that the frequency of the T allele did not differ between the women with a history of RPL and control fertile women [17]. However, the association between the C/T polymorphism or FXII activity and RPL could not be clearly elucidated, because the sample size was relatively small.

Thus, we conducted this cross-sectional and cohort study to determine the clinical significance of C/T polymorphism and FXII activity. We examined the association between 46C/T polymorphism and RPL, and between FXII activity and RPL in the cross-sectional study. We examined whether 46C/T polymorphism or FXII activity influenced the subsequent miscarriage rate in the cohort study. This was the first study to investigate the influence of *FXII* SNP on the subsequent pregnancy outcome.

## Materials and Methods

### Patients and controls

All patients were seen at Nagoya City University Hospital between September 2008 and July 2012. The study group consisted of 279 Japanese women with two or more consecutive pregnancy losses.

All patients underwent systematic examination, including hysterosalpingography, chromosome analysis of both partners, determination of aPL, including lupus anticoagulant (LA), by 5x-diluted aPTT, diluted Russel's viper venom time (RVVT) and β2 glycoprotein I-dependent anticardiolipin antibody determination (β2GPI-aCL), and blood tests for hypothyroidism and diabetes mellitus, before a subsequent pregnancy [18]. Criteria for exclusion from the analyses included the presence of uterine anomalies and chromosomal abnormalities in either partner. Patients with a history of thromboembolic events, pre-eclampsia, or abruptio placentae were also not included. The plasma samples for measurement of the FXII levels were obtained from the patients during the high phase of the basal body temperature (BBT).

Nine patients were positive for LA and 8 were positive for β2GPI-aCL. Of the 17, 7 patients were diagnosed as having APS, based on the persistence of the aPLs for more than 12 weeks.

Subsequent pregnancies of all patients were followed up until February 2013. Gestational age was calculated from BBT charts. Ultrasonography was performed once a week from 4 to 8 weeks of gestation. Dilation and curettage was performed in patients diagnosed as having miscarriage. A part of the villi was cultured, and the cells were harvested after 6–22 days of cultivation for chromosomal analysis using the standard G-banding technique.

Furthermore, 100 women with at least one child and no history of infertility or miscarriage were examined as controls. The control subjects consisted of 26 medical staff and 74 patients with dysplasia of the uterine cervix recruited from Nagoya City University Hospital from April to July 2013. None of the patients or controls were receiving any medication or were pregnant at the time of the study.

### Ethics statement

This study was conducted with the approval of the Research Ethics Committee of Nagoya City University Graduate School of Medical Sciences. Each patient provided written consent after receiving a thorough explanation about the purpose of the study and the methods to be employed.

### Cross-sectional study

The FXII activity was compared between the 17 patients with aPLs and 262 patients without aPLs in the cohort with RPL. The 17 patients with aPLs were excluded from further analysis. The characteristics of the 262 patients and 100 controls are shown in Table 1.

**Table 1:** Genotype and FXII activity in 17 patients with antiphospholipid antibodies.

	LA-aPTT	LA-RVVT	β2GPI dependent aCL	genotype	XII activity (%)
LA-aPTT-positive patients	9.8	negative	negative	CT	50
	8.7	negative	negative	CT	54
	8.3	negative	negative	CT	56
	8.2	negative	negative	CT	107
	10.9	negative	negative	TT	54
	9	negative	negative	TT	50
	8.1	negative	negative	TT	57
	8	1.3	negative	TT	53
	7.4	negative	negative	TT	65
Mean (SD) value					60.7 (17.9)
β2GPI-aCL-positive patients	negative	negative	4.6	CT	111
	negative	negative	2.8	CT	116
	negative	negative	2.4	CT	92
	negative	negative	2	CT	153
	negative	negative	10.7	TT	54
	negative	negative	7.6	TT	58
	negative	negative	5.4	TT	63
	negative	negative	2.3	TT	51
Mean (SD) value					87.3 (37.0)

10–90^th^ percentile of FXII activity according to CC, CT and TT genotype were 101–141, 72–120 and 46–77.

The allele frequencies of the CC, CT, TT genotypes of the *FXII* gene and FXII activity were compared between the 262 patients and 100 controls. We analyzed the data separately for the patients with a history of 3 or more early miscarriages before 10 weeks of gestation and those with a history of intrauterine fetal death (IUFD) in this cross-sectional study.

### Cohort study

In the present cohort, the subsequent miscarriage rate was compared among the untreated 101 patients with a history of 3 or more early miscarriage with the CC, CT or TT genotype and according to the FXII activity.

A total of 39 of the 262 patients received heparin plus aspirin or aspirin alone in deference to the patient's wishes even after she has been provided information that aspirin or heparin had, in general, no effect on the live birth rate in cases of unexplained recurrent miscarriage [19]. These patients were excluded from the cohort study.

### Statistical Analyses

The FXII activity between patients with and without aPLs was compared by student t-test.

The FXII activity was compared according to the genotype by one-way analysis of variance (ANOVA) and Tukey's multiple comparison test. The allele frequency was compared by the chi-squared test. Pearson's correlation coefficient was calculated between the aPTT and FXII activity, and between the age and FXII activity.

Multivariate logistic regression analyses were performed to examine the association of subsequent miscarriage, after adjusting for age and the number of previous miscarriages. FXII activity levels were categorized as high, normal or low using the 90^th^ percentile, 95^th^ percentile and 99^th^ percentile of the values in the controls. Furthermore, the FXII activity levels were also was categorized as high, normal and low according to the CC, CT and TT genotype using the 90^th^ percentile of the values in the controls. The FXII activity levels were also categorized into quartiles of the patients.

For the post-hoc power analysis of the genotype frequency, we used a total of 362 subjects at an α of 0.05.

All the analyses were carried out using the statistical software SPSS, Version 21. P<0.05 was considered to denote statistical significance.

### Genetic Analysis

Venous blood samples were collected in tubes containing K2 ethylenediamine tetraacetic acid and applied to genomic DNA extracting columns (QIAmp DNA blood Midi; Qiagen, Tokyo, Japan) according to the manufacturer's protocol. Polymerase chain reaction (PCR) was performed on genomic DNA samples using a Phusion High-Fidelity DNA Polymerase (NEW ENGLAND BioLabs, Finland). One µL (about 10 ng) solution (DNA preparation) was used as a template for the PCR. Exon 1 of the *FXII* gene was amplified by PCR using the sense and antisense primers 5′ CCAGTCCCACTATCTAGAAAAG-3′ and 5′ ATGGCTCATGGCTGTGATAG-3′, respectively. After initial denaturation at 98°C for 30 seconds, 35 cycles (98°C for 10 seconds, 61.9°C for 30 seconds, and 72°C for 15 seconds) and final extension at 72°C for 5 minutes were used to amplify 369-base pair products.

The substitution of 46C to T substitution is located 4 bases upstream from the translation initiation codon ATG, a region corresponding to the CseI (New Engrand BioLabs, Beverly, MA) restriction site (GACGC), which is therefore destroyed (46T). To analyze the polymorphism by electrophoresis, the samples were separated on 2% agarose gels (Takara, Japan) after enzyme digestion and stained with ethidium bromide.

To confirm the genotype, purified templates were sequenced with the BigDye Terminator v3.1 Cycle Sequencing kit (ABI Prism, Applied Biosystems, Foster City, CA, USA) on a 3100 automated sequencer.

### Factor XII activity

Plasma samples were prepared in tubes containing 3.2% sodium citrate by centrifugation at 4°C at 3,000 rpm for 10 minutes. The plasma samples were then stored at −40°C until use. The FXII activity was determined by a clotting assay using coagulation factor XII kits (HemosIL, Instrumentation Laboratory, USA). A major domestic laboratory company performed the FXII activity measurements. The intra-assay CV for the high activity control was 2.98%, and that for the low activity control was 4.03%.

## Results

Nine patients were positive for LA-aPTT and one was positive for both LA-aPTT and LA-RVVT (Table 1). The FXII activity in patients with LA was significantly lower than in the patients without LA (60.7±17.9% vs. 83.4±29.3%; p = 0.02). FXII activity in 8 patients with β2GPI-aCL alone was 87.3±37.0 (not significantly different). Thus, we excluded the 17 patients with aPLs from the following comparison.

The mean age of the fertile controls was higher than that of the patients (p = 0.002, Table 2). Eleven patients gave a history of previous intrauterine fetal death (IUFD). Secondary RPL was 18.7%.

**Table 2:** Characteristics of 262 patients with a history of recurrent pregnancy losses and 100 control healthy women with a history of live birth.

	Controls	2 or more	3 or more
No. of patients	100	262	121
Mean age (SD)	35.2 (4.7) 21–42	33.5 (4.6) 21–45	33.8 (4.8) 21–45
Mean (SD) No. of previous miscarriages	0	2.58 (0.79)	3.26 (0.69)
2		141	-
3		98	98
4		19	19
5		1	1
6		2	2
8		1	1
Mean (SD) No. of previous intrauterine fetal death	0	0.05 (0.23)	0.04 (0.24)
0		251 (95.8%)	117 (96.7%)
1–2		11 (4.2%)	4 (3.3%)
Mean (SD) No. of previous live births	1.6 (0.7)	0.20 (0.44)	0.26 (0.48)
Primary	0	213 (81.3%)	91 (75.2%)
Secondary	100	49 (18.7%)	30 (24.8%)

The results of the cross-sectional study are shown in Table 3. The wild-type (CC), heterozygote (CT) and homozygote (TT) alleles for the *FXII* gene were observed in 22 (8.4%), 139 (53.1%) and 101 (38.5%) patients with RPL vs. 17 (17.0%), 38 (38.0%) and 45 (45.0%) controls. The frequency of CT in the patients with RPL was significantly higher than that in the controls (OR, 2.83; 95% CI, 1.37–5.85; p = 0.005). The frequency of TT in the patients also tended to be higher than that in the controls. The statistical power for the frequency of CT was sufficient (1−β = 0.79), while that for the frequency of TT was insufficient (1−β = 0.31). The frequency of CT in the patients with 3 or more early miscarriage tended to be higher than that in the controls (OR, 2.32; 95% CI, 0.98–5.49; p = 0.06).

**Table 3:** **Cross-sectional study:** The frequencies of the CC, CT and TT genotypes and the factor XII activities in the 262 patients and 100 controls.

	The frequencies of the CC, CT and TT genotypes						
Genotype	Control n (%)	Patients with 2 or more miscarriage		Patients with 3 or more early miscarriage		Patients with a history of IUFD	
Total	100	262	OR (95% CI)	117	OR (95% CI)	11	OR (95% CI)
CC	17 (17)	22 (8.4)	reference	11 (9.4)	reference	0	reference
CT	38 (38)	139 (53.1)	**2.83 (1.37–5.85) 0.005**	57 (48.7)	2.32 (0.98–5.49) 0.056	8 (72.7)	-
TT	45 (45)	101 (38.5)	1.73 (0.84–3.58) 0.136	49 (41.9)	1.68 (0.71–3.98) 0.235	3 (27.3)	-
CT, TT	83 (83)	240 (91.6)	**2.23 (1.13–4.41) 0.021**	106 (90.6)	1.97 (0.88–4.44) 0.100	11 (100)	-
C/T ratio	0.36/0.64	0.34/0.66		0.34/0.66		0.36/0.64	
	The factor XII activities: mean (SD) value and range						
Total XII activity	83.8 (28.6) 40–143	83.8 (29.1) 37–178		80.9 (29.0) 37–145		71.8 (14.4) 51–94	
CC	123.1 (14.3) 97–143	126.1 (17.7) 87–160		125.2 (14.1) 97–143		-	
CT	94.3 (18.5) 55–129	89.2 (25.8) 37–178		89.9 (22.4) 37–145		74.5 (12.5) 61–94	
TT	60.0 (14.5) 40–115	65.8 (21.6) 37–139		61.6 (17.8) 37–137		64.7 (19.5) 51–87	

In regard with the C/T ratio, the frequency of T allele in the patients was similar to that of controls.

The differences in the activity levels between the CC and CT (p<0.001), CT and TT (p<0.001) and CC and TT (p<0.001) genotypes in the patients, and between the CC and CT (p<0.001), CT and TT (p<0.001) CC and TT (p<0.001) genotypes in the controls were significant. ANOVA revealed no significant difference in the mean FXII activity levels between the patients and controls. Twenty-seven of the 262 (10.3%) patients and 10 of the 100 (10%) controls showed decreased FXII activity (<50%), the difference in the percentage not being significant. A weak correlation coefficient between the aPTT and FXII activity was observed (r = −0.479). There was no correlation between the FXII activity and age.

The subsequent miscarriage rate in patients with RPL was 26.0% (68/262). A total of 32 (47.1%) miscarried conceptuses could be karyotyped, of which, 15 (46.9%) had a normal karyotype and 17 (53.1%) had an abnormal karyotype.

Association between the subsequent miscarriage rate and the *FXII* genotype or FXII activity in the 101 untreated patients with a history of RM was analyzed (Table 4). The miscarriage rates were 30.0%, 21.2% and 30.8% in the patients with the CC, CT and TT genotypes, respectively. According to the results of the logistic regression analysis, there was no increase in the miscarriage rate associated with the presence of CT and TT as compared to that associated with the presence of CC. A similar result was obtained after excluding cases with miscarriage caused by an abnormal embryonic karyotype.

**Table 4:** **Cohort study:** Subsequent miscarriage rate according to the genotype and FXII activity in 101 untreated patients with a history of recurrent miscarriage.

		Miscarriage rate	Crude analysis		Multivariable Logistic regression		Miscarriage rate excluding abnormal embryonic karyotype	Crude analysis		Multivariable Logistic regression	
			OR ^a^ (95% CI ^b^)	P-value	OR (95% CI)	P-value		OR (95% CI)	P-value	OR (95%CI)	P-value
Genotype	CC	30.0% (3/10)	reference		reference		22.2% (2/9)	reference		reference	
	CT	21.2% (11/52)	0.40 (0.08–1.96)	0.26	0.40 (0.07–1.96)	0.26	19.6% (10/51)	0.86 (0.15–4.76)	0.86	0.50 (0.08–3.06)	0.46
	TT	30.8% (12/39)	1.04 (0.23–4.72)	0.96	0.79 (0.17–3.73)	0.77	20.6% (7/34)	0.90 (0.15–5.38)	0.92	0.68 (0.11–4.18)	0.68
FXII activity (10–90^th^ percentile)^c^	Normal	31.3% (22/83)	reference		reference		20.8% (16/77)	reference		reference	
	High	37.5% (3/8)	1.66 (0.37–7.52)	0.51	1.96 (0.41–9.35)	0.40	28.6% (2/7)	1.52 (0.27–8.62)	0.63	1.84 (0.31–10.99)	0.50
	Low	10.0% (1/10)	0.29 (0.04–2.57)	0.28	0.35 (0.04–3.01)	0.34	10.0% (1/10)	0.42 (0.05–3.60)	0.43	0.50 (0.06–4.31)	0.52
Genotype and FXII activity (10–90^th^ percentile)^d^	Normal	25.3% (19/75)	reference		reference		21.1% (15/71)	reference		reference	
	High	66.7% (6/9)	**5.88 (1.34–25.64)**	**0.02**	**5.65 (1.24–25.64)**	**0.03**	50.0% (3/6)	7.75 (0.68–20.41)	0.28	4.22 (0.73–24.4)	0.11
	Low	5.9% (1/17)	0.18 (0.02–1.48)	0.11	0.20 (0.02–1.62)	0.13	5.9% (1/17)	0.23 (0.03–1.90)	0.17	0.24 (0.03–2.07)	0.24
FXII activity (quartile)	−56	10.3% (3/29)	reference		reference		7.1% (2/28)	reference		reference	
	57–84	31.8% (7/22)	4.05 (0.91–18.18)	0.07	3.60 (0.78–16.40)	0.10	25.0% (5/20)	4.33 (0.75–25.00)	0.10	3.86 (0.64–23.26)	0.14
	85–101	36.0% (9/25)	**4.88 (1.15–20.83)**	**0.03**	**4.67 (1.06–20.41)**	**0.04**	33.3% (8/24)	**6.49 (1.22–34.48)**	**0.03**	**6.37 (1.15–34.48)**	**0.03**
	102–	28.0% (7/25)	3.37 (0.77–14.71)	0.11	3.23 (0.71–14.49)	0.13	18.2% (4/22)	2.89 (0.48–17.54)	0.25	2.76 (0.44–17.54)	0.28

a; odds ratio, b; confidence interval, c; Normal 50–127, High 128-, Low -49,

d; CC 101–141, CT 72–120, TT 46–77.

The logistic regression analysis also showed no significant increase in the miscarriage rate associated with low or high FXII activity levels, classified using the cutoff values of 10–90^th^ percentile, 5–95^th^ percentile and 1–99^th^ percentile of the control values (Table 4). The frequencies obtained using the latter two cutoffs seemed to be insufficient to detect any significant differences (not shown). The multivariable analyses showed no significant increase in the miscarriage rate associated with FXII activity levels even after excluding cases with an abnormal embryonic karyotype.

Furthermore, when the FXII activity was categorized into high, normal or low classified using the 10–90^th^ percentile of the control values for each of the CC, CT and TT genotypes, the logistic regression analysis showed significant increase in the miscarriage rate associated with high FXII activity level (OR, 5.65; 95% CI, 1.24–25.64; p = 0.03). However, the difference disappeared after exclusion of cases with an abnormal embryonic karyotype. When the FXII activity was categorized into quartiles, the intermediate level, that is, a FXII activity level of 85–101%, predicted subsequent miscarriage (OR, 4.65; 95% CI, 1.06–20.4; p = 0.04). The result remained significant after excluding cases with an abnormal embryonic karyotype.

## Discussion

The FXII activity in the 9 patients with LA was significantly lower than that in the patients without LA in the present study. This was in line with the results of Gallimore's study [20]. LA might include antibodies to factor XII, as an association has been reported between the presence of antibodies to factor XII and recurrent fetal loss in patients with APS [21]. In the present study, there was no difference in the FXII activity levels between the patients and controls after patients with LA were excluded.

In our previous study, we failed to show an association between LA and reduced FXII activity levels because we compared only patients with the TT genotype [17]. We previously reported that low FXII activity, but not an associated common genetic polymorphism, 46C/T, was linked to RPL [17]. A systematic review concluded that FXII deficiency is associated with RM [22]. Pauer et al. reported that 10 of 67 women with primary recurrent abortion (14.9%) and 4 of 33 women (12.1%) with secondary recurrent abortion had reduced factor XII activity (<60%), while all controls had normal FXII activity [23]. However, they checked for only anticardiolipin antibody, and not LA. These results could be attributable to the inclusion of patients with LA.

Low levels of FXII was confirmed not to influence the subsequent miscarriage rate when the cutoff values were based on the 90^th^ percentile, 95^th^ percentile and 99^th^ percentile of the control values. We previously reported that the miscarriage rate in patients with low FXII activity levels (less than 39%) was significantly higher than that in the patients with normal activity levels [16]. The sample size in our previous study was relatively small, because 4 of the 5 untreated patients with FXII activity levels of less than 39% developed miscarriage. The present study included the largest sample size, and was the first prospective cohort study.

CT genotype, but not the TT genotype was confirmed to be a risk factor for RM. We found no association between 46C/T polymorphism and RPL in a previous study, because the frequency of this SNP was similar between the patients and controls (0.31/0/69 and 0.31/0.69) [17]. We did not conduct comparisons according to the genotype distribution, although the frequencies of CC, CT and TT in the patients and controls were 9.6%, 43.4% and 47.0% and 16.4%, 29.9% and 53.7%, respectively, in our previous study, being quite similar to those in the present study.

Walch et al. demonstrated that the genotype distribution was not significantly different between a RM group of 212 patients and a control group of 149 women in Middle-European Caucasian population [24]. It is speculated that the distribution of the risk alleles might show ethnic differences.

Johnson et al. found a very weak association between myocardial infarction and the CT+TT genotype (OR 1.13, 95% CI 1.00–1.27), and suggested that this was caused by low FXII level activity because of the T allele [25]. We found a similar association between the CT+TT genotype and RPL (Table 3), however, only CT was significant, not TT. The statistical power for the frequency of CT was sufficient (1−β = 0.79). The statistical power for the frequency of TT was insufficient and (1−β = 0.31). This implies that a larger study might have possibilities to show the significant of differences for the TT genotype.

That the T allele might act via other mechanism(s), such as endothelial dysfunction, and not via low FXII activity, to induce myocardial infarction and miscarriage. LaRusch found that FXII initiates signaling to induce human umbilical vein endothelial cell proliferation, growth and angiogenesis [26]. FXII as a growth factor stimulates angiogenesis after ischemia, inflammation, and injury, just like vascular endothelial growth factor. FXII plays various roles in vivo: FXII triggers the plasma contact system via the kallikrein kinin-system and intrinsic coagulation pathway, and zymogen FXII functions as a growth factor that mediates cell signaling leading to proliferation and stimulation of angiogenesis. FXII might have another role of maintaining pregnancy.

Low levels of FXII did not increase the subsequent miscarriage rate when the cutoff values were based on the 90^th^ percentile, 95^th^ percentile and 99^th^ percentile of the control values. Activity levels of 85–101%, which fall in the second highest quartile, and not low FXII activity levels, were found to predict subsequent miscarriage. This intermediate range might correspond to the CT genotype, because the reported mean (SD) FXII activity in patients with the CT genotype is 89.2 (25.8), even though the CT genotype itself did not influence the likelihood of subsequent miscarriage.

Many cross-sectional studies have been reported on associations between polymorphism and RPL, such as 4G/4G for plasminogen activator inhibitor-1 polymorphisms (OR 11.0, 95% CI 2.3–52.4), protein Z intron F G79A polymorphism (OR 0.3, 95% CI 0.1–0.8) and Annexin A5's -1C/T (OR 2.7, 95% CI 1.0–6.7) [27]–[29]. However, the clinical significance could not be established because most were not cohort studies. Our cross-sectional study confirmed that variations in the *ANXA5* gene upstream region, especially SNP5, were risk factors for RPL, and our cohort study concluded that the presence/absence of the *ANXA5* risk allele did not have any significant predictive effect on the subsequent pregnancy outcome [30].

FXII activity level in the intermediate range, that is, 85–101% was predictive of subsequent miscarriage. The FXII activity is increased in old age, in females, during pregnancy, during intake of an oral contraceptive. The influence of age could be ignored, although all the patients were younger than the control women, because there was no correlation between the FXII activity and age in the present study. We could not measure FXII activity in duplicate. These are some limitations of the present study. Further study is needed because this was a new finding.

The CT genotype of the *FXII* gene was confirmed to be a risk factor for RPL, but it was not shown to serve as a reliable clinical predictor of the subsequent pregnancy outcome. Therefore, we propose that testing for this allele is not needed, as it is without clinical benefit and is an unnecessary expense.
